# HIV-1 Gag recruits oligomeric Vpr via two binding sites in p6, but both mature p6 and Vpr are rapidly lost upon target cell entry

**DOI:** 10.1128/JVI.00554-21

**Published:** 2021-08-10

**Authors:** Madushi Wanaguru, Kate N. Bishop

**Affiliations:** a Retroviral Replication Laboratory, The Francis Crick Institute, London, NW1 1AT, United Kingdom

## Abstract

The p12 region of MLV Gag and the p6 region of HIV-1 Gag contain late-domains required for virus budding. Additionally, the accessory protein Vpr is recruited into HIV particles via p6. Mature p12 is essential for early viral replication events, but the role of mature p6 in early replication is unknown. Using a proviral vector in which the *gag* and *pol* reading frames are uncoupled, we have performed the first alanine-scanning mutagenesis screens across p6, to probe its importance for early HIV-1 replication and to further understand its interaction with Vpr. The infectivity of our mutants suggests that, unlike p12, p6 is not important for early viral replication. Consistent with this, we observed that p6 is rapidly lost upon target cell entry in time-course immunoblotting experiments. By analysing Vpr incorporation in p6 mutant virions, we identified that the 15-FRFG-18 and 41-LXXLF-45 motifs previously identified as putative Vpr-binding sites are important for Vpr recruitment, but that the 34-ELY-36 motif also suggested to be a Vpr-binding site is dispensable. Additionally, disrupting Vpr oligomerization together with removing either binding motif in p6 reduced Vpr incorporation ~25-50-fold more than inhibiting Vpr oligomerization alone and ~10-25-fold more than deletion of each p6 motif alone, implying that multivalency/avidity is important for the interaction. Interestingly, using immunoblotting and immunofluorescence, we observed that most of Vpr is lost concomitantly with p6 during infection, but that a small fraction remains associated with the viral capsid for several hours. This has implications for the function of Vpr in early replication.

## Introduction

**Figure 1:**
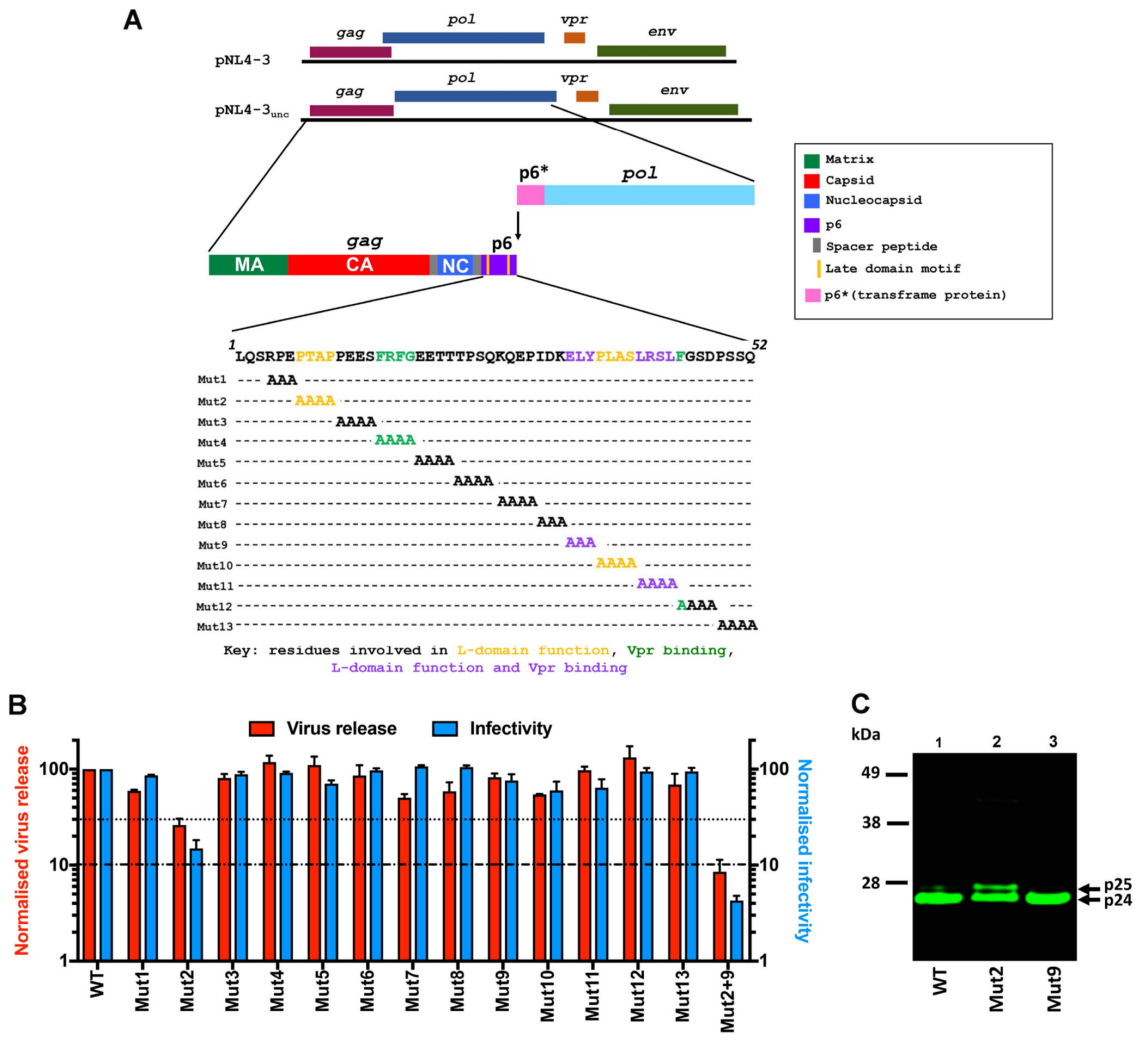
HIV-1 p6 has no essential functional roles in early infection. A) Schematic representation of the gag, *pol, vpr and env* open reading frames in WT pNL4- 3 and in the pNL4-3_unc_ proviral plasmid (27) used in this study. In pNL4-3_unc_, the pol frame no longer overlaps with the p6 region of gag. The arrow indicates the frameshift signal at the 3’ end of gag. The p6 amino acid sequence from pNL4-3_unc_ is also shown, with the alanine-scanning mutants 1-13 used in this study annotated. Coloured residues indicate those reported to recruit ESCRT proteins (yellow), the viral accessory protein Vpr (green) or both (purple). B) Measurement of virus release and single-cycle infectivity of p6 mutants, including the combinatorial L-domain mutant (2+9). 293T cells were transfected with equal volumes of WT pNL4-3_unc_ or mutant HIV-1 pNL4-3_unc_ constructs. Viruscontaining culture supernatants were harvested 48 h post-transfection and titres were estimated using a RT ELISA assay as a proxy for virus release (red bars). HeLa TZM-bl cells were then challenged with equivalent RT units of WT or mutant virus. Infectivity was measured 48 h post-challenge by detection of beta-galactosidase activity in a chemiluminescent reporter assay (blue bars). The data are plotted as percentage of WT virus release and infectivity (mean ± SEM of 3 biological replicates). C) Immunoblot showing CA-Sp1 processing efficiency of WT and selected p6 mutants. Virus particles were concentrated by ultracentrifugation through a sucrose cushion and then equivalent RT units analysed by SDS-PAGE and immunoblotting using an anti-CA antibody.

The retroviral Gag polyprotein and its cleavage products are essential for multiple stages of the viral replication cycle. In late replication, Gag directs the assembly and release of progeny viral particles. Then, during virion maturation, orthoretroviral Gag is proteolyically cleaved to generate a number of separate proteins including the three main structural proteins, matrix (MA), Capsid (CA), and nucleocapsid (NC) (Figure 1A). In addition, many retroviruses also produce other Gag cleavage products, usually named for their apparent molecular weight, such as the p12 protein of MLV and the p6 protein of HIV-1 (1). In many retroviral genera these additional products are situated between MA and CA, although the p6 protein of HIV is found at the end of Gag. Regardless of their size or location in Gag, these additional cleavage products usually contain the proline-rich late (L)-domains which recruit components of the cellular endosomal sorting complex for transport (ESCRT) machinery required for efficient virus budding (2-5). Significantly, MLV p12 has also been found to be essential for reverse transcription and integration, commonly referred to arbitrarily as early post-entry replication events, with roles in viral core stabilisation and nuclear retention/chromatin targeting (6-13). Functional characterisation of HIV-1 p6, however, has so far been primarily restricted to its roles in the context of Gag and the involvement, if any, of mature p6 in early replication events remains unknown.

Much of the characterisation of MLV p12 has been performed using systematic and comprehensive mutagenesis screens (6, 7). HIV-1 p6 on the other hand has had limited scope for such analysis because its reading frame overlaps with that of *pol* in the native configuration. Additionally, mutations in p6 can potentially impact the efficiency of ribosomal frameshifting, a key feature of HIV-1 Gag-Pol translation. Due to these restrictions, functional characterisation of HIV-1 p6 has been predominantly reliant on using recombinant proteins and chimearic viruses.

Studies on HIV-1 p6 have so far uncovered two important functional roles of this region of Gag during the late stages of virus replication. Apart from recruiting the ESCRT proteins TSG101 and ALIX via the two L-domain motifs, P(T/S)AP and LYPXnLXXL, respectively (1, 2, 14-18) (Figure 1A), HIV-1 p6 also mediates the incorporation of the accessory protein, Vpr, into budding virion assemblies (19-25). However, many aspects of the HIV-1 p6-Vpr interaction remain to be elucidated. For example, there is inconsistency in the literature about the precise regions of p6 involved in binding Vpr and their relative contribution (20, 23-25). Moreover, whether the interaction between p6 and Vpr persists following virion maturation and if it influences Vpr function are also unknown (21).

This *status quo* of HIV-1 p6 research has however recently been transformed by the design and utilisation of proviral vectors in which the *gag* and *pol* reading frames are uncoupled (26, 27). By repositioning the slippery sequence in p6 (that induces the frame shift that creates *gag-pol)* to the end of *gag* and duplicating part of the p6 sequence that is normally read in the *pol* reading frame, called p6*, the p6 sequence no longer overlaps with *pol*, and can be mutated without affecting the *pol* gene. This proviral contruct therefore expresses a Gag protein with an extra five residues on the end and a Gag-Pol poly-protein that contains p6 fused directly to the transframe protein, p6*. All other proteins, including Vpr, are expressed from this provirus as normal. Here, using this vector previously reported in Radestock *et al* (27), we have performed the first alanine-scanning mutagenesis screens across the whole of p6, both to investigate its involvement in the early stages of replication and to gain more mechanistic insight into its interaction with Vpr. By analysing HIV-1 p6 mutants for infectivity, we have found that, in contrast with MLV p12, p6 is unlikely to be essential for early replication. In agreement with this, we observed that mature p6 is very rapidly lost upon target cell entry in time-course immunoblotting experiments. Additionally, our analysis of Vpr incorporation by p6 mutants suggests the presence of two seemingly equivalent Vpr-binding sites in the p6 region of Gag (a third proposed binding site was dispensable for Vpr packaging). We have investigated the effects of mutating these regions in a Vpr oligomerization mutant and have assimilated our findings into an avidity-based multivalent binding model for the p6- Vpr interaction. Furthermore, similarly to p6, we also saw a very rapid and significant loss of viral Vpr following host cell infection in our immunoblotting assays. Importantly however, based on our observations of Vpr localisation from immunofluorescence, the residual protein which persists seems to remain associated with viral CA during the first 4 hours of infection.

## Results & Discussion

### HIV-1 p6 is not required for early replication events in cell culture

In addition to recruiting the ESCRT machinery in late replication via its L-domain motif, the MLV p12 protein performs essential functions during the early stages of infection (6, 7). Our lab recently established that a motif towards the N-terminus of MLV p12 directly binds and stabilises the CA shell of the virus during early infection (7, 11). Work by ourselves and others also revealed that a motif towards the C-terminus of the protein tethers the CA-containing viral pre-integration complex to mitotic chromatin by directly interacting with nucleosomal histones (Figure 1A) (8-10, 12, 13). In HIV-1, the only additional Gag cleavage product is p6, and it is the p6 region of Gag that contains the L-domain. To investigate whether, like MLV p12, mature HIV-1 p6 is also essential for early replication, we embarked on a systematic mutational analysis of the protein.

We used a previously reported NL4-3-based HIV-1 proviral plasmid in which the *gag* and *pol* reading frames are uncoupled, pNL4-3_unc_ (27), and generated a series of 13 alanine-substitution mutants in p6 (Figure 1A). We substituted all p6 residues apart from the first three, which are essential for p6 cleavage from Gag. Three or four residues were changed to alanine in each mutant, spanning the length of p6, including the L-domain motifs (Figure 1A, yellow and purple residues) and sequences identified as Vpr-binding sites in previous studies (Figure 1A, green and purple residues) (20, 23-25).

Viruses carrying WT or mutant p6 Gag were produced by transfection of 293T cells, and cell supernatants were analysed using a modified ELISA for reverse transcriptase (RT) activity to determine the viral titres. Interestingly, we observed similar virion release to WT HIV-1 with all mutants apart from Mutant 2, which only produced ~30% of the WT titre (Figure 1B, red bars). In Mutant 2, the N-terminal L-domain, PTAP, which is known to bind to the ESCRT-1 protein TSG101 in late replication (18, 28, 29) was replaced with alanines. Residues which constitute the second L-domain of p6, which recruits the ESCRT-associated protein ALIX, were substituted in Mutants 9, 10 and 11 of our panel (17, 30, 31). However, none of these C-terminal L-domain mutants displayed appreciable defects in viral release. A combinatorial mutant, carrying the alanine substitutions of both Mutant 2 and 9, did however show a further >3-fold reduction in viral release compared to the reduction seen with Mutant 2 alone (Figure 1B). Our results therefore suggest that in 293T cells, binding to ALIX is mostly important for virus release in the absence of TSG101 recruitment. This observation of the functional dominance of P(T/S)AP over LYPXnLXXL is consistent with reported findings in both 293T and other cell lines (2, 31-34). Furthermore, no other regions in p6 appear to be essential for late replication events.

To investigate the function of p6 in early replication events, TZM-bl HeLa cells were challenged with equivalent RT units of the mutant viruses in single-cycle infectivity assays. TZM-bl HeLa reporter cells express HIV receptors and a Tat-inducible LacZ gene, allowing beta-galactosidase activity to be assayed as a measure of HIV infection. Interestingly, only Mutant 2 showed any significant defect in infection, attaining ~20% of WT infectivity (Figure 1B, blue bars). As well as decreasing the efficiency of viral release, defects in TSG101 binding are known to impede/delay Gag processing during virion maturation, leading to the accumulation of the CA-SP1 (p25) processing intermediate (2, 27). Indeed, in agreement with previous studies, immunoblotting analysis of our viral lysates revealed a higher p25:p24 CA ratio of Mutant 2 compared to WT and Mutant 9 (Figure 1C). Therefore, based on current understanding, the infectivity defect of Mutant 2 likely stems from impaired mature conical core assembly due to the dominant-negative effect exerted by the presence of increased levels of Gag processing intermediates (35-38). Importantly, the high levels of infectivity of the rest of the alanine-scanning mutants suggests that, unlike MLV p12, HIV-1 p6 is not essential for the early stages of virus replication in cell culture.

### HIV-1 p6 binds Vpr via two binding sites in late replication

**Figure 2:**
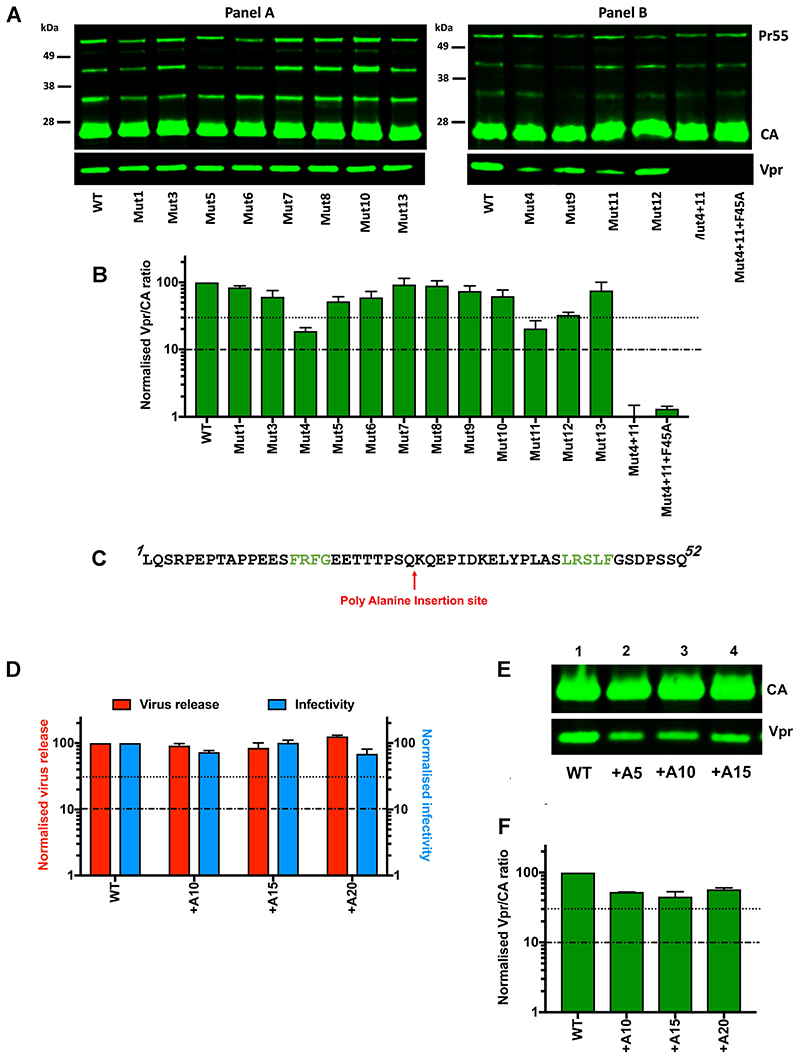
HIV-1 p6 recruits Vpr via two binding sites during late infection. A) Representative immunoblots demonstrating Vpr incorporation levels of WT and mutant virus, including combinatorial p6 mutants (4+11) and (4+11+F45A). Virus particles were concentrated by ultracentrifugation through a sucrose cushion and then equivalent RT units were separated by SDS-PAGE and analysed by immunoblotting using anti-CA and anti-Vpr antibodies. B) Vpr/CA ratios were estimated by quantifying relative band intensities compared to WT virions on immunoblots such those shown in (A) using a LI-COR imager. C) Sequence of p6 showing the position of insertion of poly alanine sequences. Vpr-binding motifs are highlighted in green. D) Virus release efficiency and single-cycle infectivity of the p6 insertional mutants was analysed as in Figure 1. Histograms show mean ± SEM of 3 biological replicates. E) and F) Representative immunoblot and quantification of Vpr incorporation (Vpr/CA ratio) for the p6 insertion mutants, analysed as in (A) and (B).

The role of the p6 region of Gag (p6-Gag) in ESCRT binding has been well characterized. However, less is known about the other reported function of p6, Vpr incorporation into virions. The motifs FRFG (24), ELY (25) and LXXLF (20-23) of p6 have all been proposed as putative Vpr-binding sites, but there is currently little agreement about the relative contributions of these motifs to Vpr-binding. Therefore, to investigate which regions of p6 are involved in Vpr recruitment, we measured the Vpr incorporation levels of our p6 mutant panel by calculating the Vpr: CA ratios following immunoblot analysis of equivalent RT units of virus. Three of our panel, Mutants 4, 11 and 12, incorporated less than 30% Vpr compared to WT virions (Figures 2A and 2B). These carried alanine substitutions in the previously reported FRFG (Mutant 4) or LXXLF (Mutants 11 and 12) Vpr-binding motifs (Figure 1A). Surprisingly, Mutant 8, in which the ELY sequence had been altered, did not show any appreciable reduction in virion Vpr levels, suggesting that this motif may be dispensable. Indeed, combinatorial changes of FRFG with LXXL (Mutant 4+11) or LXXLF (Mutant 4+11+F45A), reduced virion Vpr levels by ~100-fold or more, confirming these as the primary Vpr-binding sites within p6 (Figures 2A and 2B).

As alanine substitution of both FRFG and LXXLF motifs together reduced Vpr packaging to a far greater extent than either alone, these motifs appeared to have some redundancy. To gain more mechanical insight into the interaction between p6 and Vpr, we performed further mutational analyses. Firstly, to determine whether the spacing between the two Vpr-binding sites of p6 was important, we inserted poly-alanine linkers of varying length into the middle of the protein, between the two binding motifs (Figure 2C), and tested the effect on Vpr incorporation. Interestingly, the insertion of 10, 15 or 20 alanine residues did not significantly decrease either viral fitness (Figure 2D) or Vpr incorporation (Figure 2E and F), suggesting that the spacing of the binding sites was not critical and that each site may be acting independently of the other. However, poly-alanine linkers are conformationally flexible and the insertions may not have significantly altered the positioning of the two binding sites relative to each other in 3-dimensional space.

### Virion-associated Vpr is oligomeric

Vpr is a small basic protein with a central hydrophobic core comprised of three amphipathic alpha helices (39, 40). Recombinant Vpr has been observed to self-associate both *in vitro* and in cells (21, 41, 42). Vpr oligomerization is mediated by its hydrophobic core and can be disrupted by various mutations in its alpha helices, including L67A in helix three (41, 43). Based on current evidence, Vpr oligomerization does not seem to be required for its apoptotic and cell cycle arrest functions, however, it has been proposed to be important and even essential for its interaction with Gag and recruitment into virion particles (43-45).

**Figure 3:**
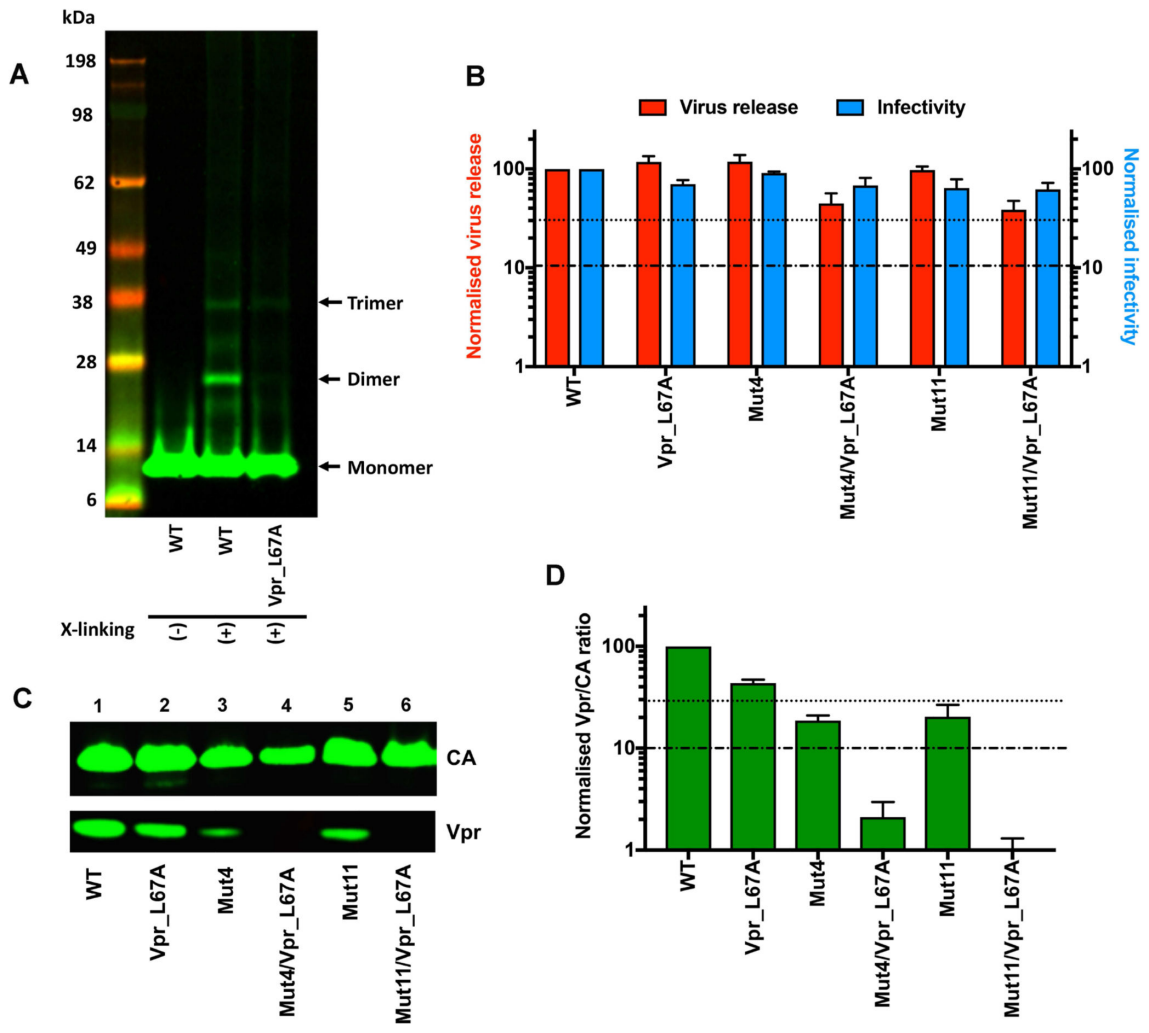
Vpr oligomerization is necessary for incorporation if one p6 binding site is removed. A) Immunoblot showing the presence or absence of Vpr oligomers in virus carrying WT or an L67A mutant Vpr. Vpr oligomers were cross-linked for analysis by fixing equivalent RT units of virus in 1% formaldehyde prior to concentration by ultracentrifugation and analysis by SDS-PAGE and immunoblotting with the anti-Vpr antibody. Possible Vpr monomers, dimers and trimers are indicated by the black arrows. B) Virus release efficiency (red bars) and single-cycle infectivity (blue bars) of virus carrying mutations in both p6 and Vpr was analysed as in Figure 1. Histograms show mean ± SEM of 3 biological replicates. C and D) Representative immunoblot and quantification of Vpr incorporation (Vpr/CA ratio) in p6/Vpr dual mutants, analysed as in Figure 2.

To investigate the role of Vpr oligomerization in the interaction with p6-Gag in our system, we first probed the oligomeric state of virion-associated Vpr by formaldehyde cross-linking. We have previously used the same protocol to demonstrate the interaction between CA and the N-terminal region of p12 in MLV virions (12). When HIV-1 particles were lysed following cross-linking and analysed by immunoblotting with an anti-Vpr antibody, we observed additional bands corresponding in size to dimers and trimers of the protein (Figure 3A). Significantly, these oligomeric forms were depleted in virion particles carrying the Vpr mutant L67A (Figure 3A).

### Vpr binding to p6-Gag requires two out of three possible interactions

After validating that Vpr oligomerization was disrupted by the L67A mutation, we then characterised the fitness and Vpr incorporation of virions carrying this mutation. In comparison with virus carrying WT Vpr, we observed no appreciable defects in either viral release or infectivity of Vpr L67A virions (Figure 3B, second pair of bars). Surprisingly, we also only detected a small, ~2-fold, reduction in Vpr incorporation in these mutant particles (Figure 3C and D, Lane 2). Our results therefore suggest that although Vpr is oligomeric in WT HIV-1, oligomerization *per se* is not essential for its interaction with p6. The discrepancy between our findings and those of some previous studies (43, 44) that concluded that Vpr oligomerization was essential for p6 binding is currently unclear, but may be due to differences in experimental design, such as using un-tagged *vs* tagged forms of Vpr and expressing Vpr from the same construct as Gag (as done here) *vs* separate plasmids.

Next, we tested the effect of altering either of the Vpr-binding sites of p6 when Vpr oligomerization was compromised. Virions carrying the Vpr L67A mutation in combination with either the p6 Mutant 4 or Mutant 11 alanine changes did not show any notable defects in viral release or infectivity (Figure 3B) but did show a significant reduction in Vpr incorporation (Figure 3C and D). The levels of Vpr packaged into these viral particles were >10-fold lower than the levels of Vpr packaged into p6 Mutant 4 or Mutant 11 virus carrying WT Vpr (Figure 3C and D).

**Figure 4:**
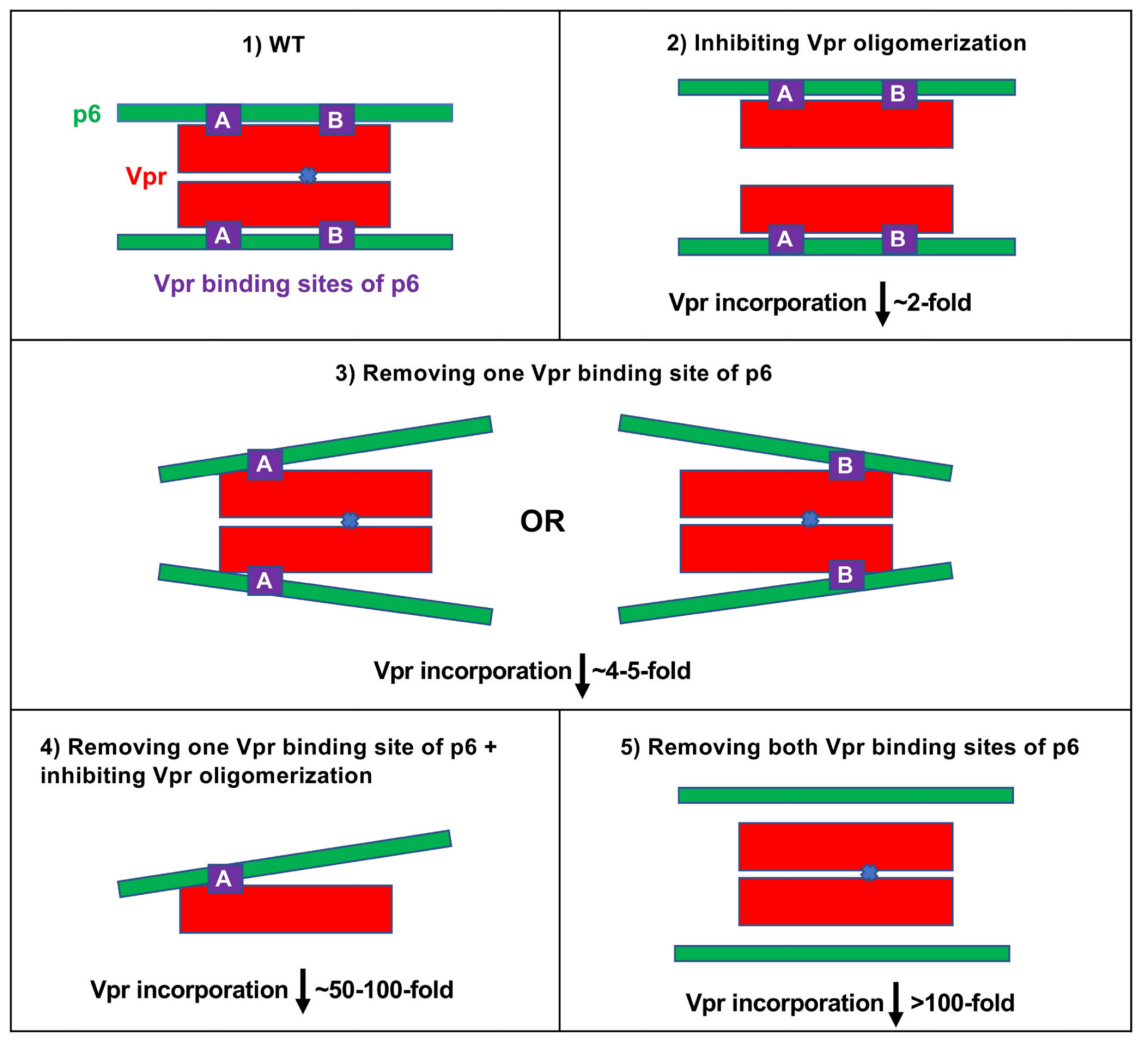
An avidity-based multivalent binding model for the HIV-1 p6-Vpr interaction. WT p6 (green bars) recruits oligomeric Vpr (red bars with purple interaction site) via two binding sites, [A] and [B] (Scenario 1). Inhibiting Vpr oligomerization decreases Vpr incorporation by ~2-fold (Scenario 2). Virion-associated Vpr levels decrease by ~4-5-fold when either of the binding sites in p6 are removed (Scenario 3). However, preventing Vpr oligomerization, together with the deletion of one Vpr-binding site in p6 decreases Vpr packaging by ~50-100 fold (Scenario 4) and deleting both binding sites in p6 decreases Vpr incorporation >100-fold (Scenario 5).

Overall, our results suggest that both the oligomerization of Vpr and the presence of two Vpr-binding sites in p6 are required for maximal Vpr recruitment. Inhibiting the interaction at any one of these binding sites results in a 2-5 fold reduction in Vpr incorporation, but simultaneously removing two binding sites results in >100 fold reduction in Vpr packaging (Figure 4). We postulate this to be indicative of the reliance of the p6-Vpr interaction on avidity arising from multivalency.

### Virion-associated p6 and Vpr are rapidly lost during host cell entry

Following maturation, the p6 protein will be present in viral particles. However, whether it remains with the viral core and/or bound to Vpr following infection has not been investigated. Vpr has been reported to remain associated with the viral core throughout the early stages of replication, and exogenously expressed Vpr-GFP has been used as a marker for HIV-1 cores (46, 47). However, how it remained associated with the core was not addressed, and a recent study has postulated that Vpr in fact decouples from the core soon after host cell entry and migrates to the nucleus, like its paralogue Vpx (48). The function of Vpr is currently hotly debated and is likely influenced by its localisation and links to the viral core. Therefore, to investigate changes in both mature p6 and Vpr levels and localisation during early host cell infection, we performed a series of immunoblotting and immunofluorescence-based time course experiments using pNL4-3_unc_ virus and infection of TZM-bl HeLa cells.

**Figure 5:**
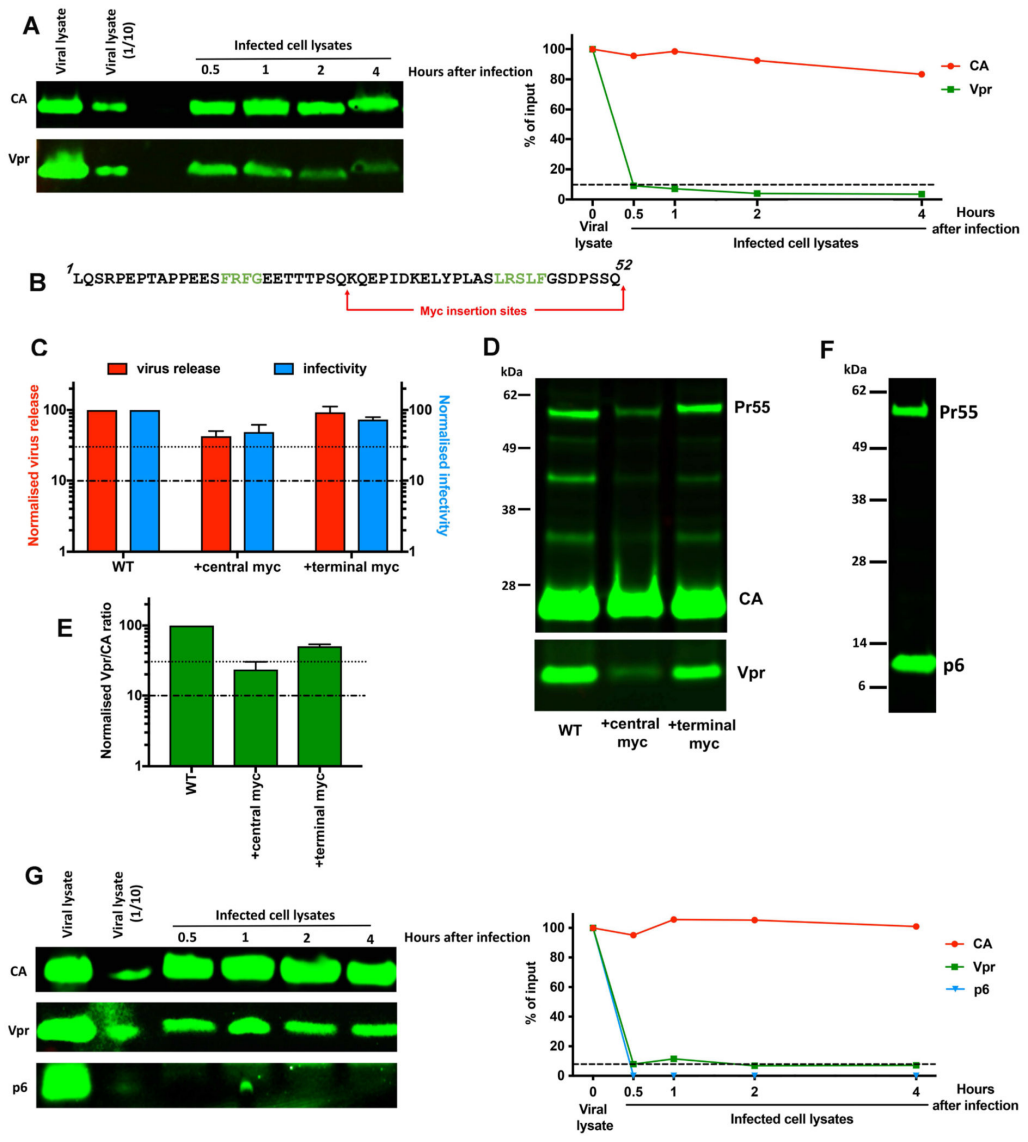
There is a rapid loss of HIV-1 p6 and Vpr compared to CA during early infection. Time-course assays were performed to monitor changes in CA, Vpr and p6 levels in infected cells. After infection by spinoculation with virus carrying WT (A) or myc- tagged (G) p6, HeLa TZM-bl cells were incubated at 37°C for 30 min before replacement of media with fresh DMEM. Infected cells were subsequently harvested at different time points and lysed and analysed by immunoblotting with (A) anti-CA and anti-Vpr and (G) anti-CA, anti-Vpr and anti-myc antibodies. Cell lysates were analysed alongside viral lysate prepared from the input virus stock for comparison. Relative viral protein levels were estimated by quantifying immunoblot band intensities and plotted as % of input. B) Sequence of p6 showing the position of myc tag insertion sites. Vpr binding motifs are highlighted in green. C-E) Virus release efficiency, single-cycle infectivity and Vpr incorporation efficiency (Vpr/CA ratio) of virus carrying myc-tagged p6 were analysed as in Figure 2. The data in histograms are plotted as percentage of WT virus (mean ± SEM of 3 biological replicates). F) Immunoblot analysis with anti-myc antibody of viral lysates from viruses carrying C-terminally myc-tagged p6.

Firstly, we synchronously infected TZM-bl cells with WT virus and harvested cells at different time points to analyse viral protein levels in whole cell lysates by immunoblotting using anti-CA, anti-Vpr and anti-p6 antibodies. Viral lysate prepared from the input virus stock was used as a control for initial protein levels, and relative protein levels were estimated for each time point by quantifying immunoblot band intensities compared to this control. Interestingly, whereas there was only a minor reduction in total CA levels in the cell during the first 4 hours of infection, we saw a loss of ~90% of virion-associated Vpr compared to input virus, concomitant with host cell entry, with levels decreasing slowly thereafter at a comparable rate with CA (Figure 5A).

Unfortunately, the anti-p6 antibody used in our assay was not sufficiently sensitive or specific for robust detection of mature p6 under these experimental conditions. To try and detect viral p6 in our time-course experiments, we therefore tested the viability of inserting a myc-tag into the Gag protein either within the central region of p6, where we had previously inserted poly-alanine linkers without decreasing infectivity (Figure 2C), or at the C-terminus of the protein (Figure 5B). pNL4-3_unc_ virions carrying myc-tagged p6 did not show significant defects in particle release or infectivity with either insertion (Figure 5C) and their CA processing was similar to WT (Figure 5D). However, unlike the insertions of poly-Alanine linkers at this site, Vpr recruitment was significantly impeded by the presence of the central myc-tag (Figures 5D and E), suggesting that the myc-tag in this position interferes with Vpr-binding perhaps via structural alterations. In contrast, the C-terminal myc-tag did not reduce Vpr packaging (Figures 5D and E) and was therefore deemed suitable for our investigations. We next tested the recognition of C-terminally-myc tagged p6 by an anti-myc polyclonal on viral lysate immunoblots and observed robust detection of mature p6 as well as unprocessed Gag, Pr55 (Figure 5F). Finally, we repeated the TZM-bl infection time-course experiment with virus carrying C-terminally myc-tagged p6 and analysed the cell lysates by immunoblotting with anti-CA, anti-Vpr and anti-myc antibodies (Figure 5G). Interestingly, although we could detect p6 in the input virus lysate, we did not observe any mature p6 in infected cell lysates, suggesting that most of p6, similarly to Vpr, rapidly becomes undetectable upon host cell entry (Figure 5G).

Our immunoblotting analyses were performed on whole cell lysates. Therefore, we propose that the vast majority of Vpr and mature p6 proteins are rapidly degraded following viral entry. The absence of any detectable mature p6 in infected cells is consistent with the findings from our alanine-scanning mutagenesis screen which suggest that p6 is functionally dispensable during early viral replication. It is also consistent with Hahn and colleagues’ proposal of mature p6 being a target of the insulin degrading enzyme, a cytosolic metalloprotease (49). Interestingly, Vpr has been detected as a virion-free protein in the serum and cerebrospinal fluid of patients infected with HIV-1 (50). Extracellular Vpr concentrations have been found to correlate with plasma viremia (51). Thus, although improbable, Vpr and p6 could also potentially be exported from host cells. It is also formally possible that the reduced levels of Vpr and p6 observed in cell lysates in our immunoblotting analyses were due to these proteins being excluded from entering the polyacrylamide gel matrix as a consequence of their association with cell membranes or other insoluble cellular material. However, as cells were lysed with RIPA buffer, we think this an unlikely explanation for our results.

Nonetheless, our immunoblotting data implies that neither the majority of mature p6 nor Vpr molecules are trapped inside the viral core, which is thought to take longer than 30 minutes to uncoat, and may, in fact, remain intact until immediately prior to integration (52, 53). Previously, it has been inferred that p6 is primarily located outside of the mature core, as it is absent from detergent-extracted HIV-1 core preparations (54, 55). Additionally, the counterpart of p6 in the non-primate lentivirus equine infectious anaemia virus, p9, has also been proposed to be located outside of the virion core (56). Furthermore, in an immuno-EM study of mature and immature HIV-1 particles by Wang et al., Vpr has been observed to be primarily located immediately beneath the viral envelope rather than within the virion core (57). This is supported by an immunofluorescence study reporting rapid dissociation of Vpr from the viral core following infection (48). In this study, single particle imaging allowed tracking of individual cores with time and showed that cores lost detectable Vpr within 15-20 minutes post-fusion. The released Vpr then rapidly entered the nucleus where it formed large complexes with cellular proteins. It was not clear in this report how long the Vpr persisted in the nucleus, but as the Vpr was tagged with fluorescent proteins, this may have stabilised the protein and decreased the rate of degradation. Other studies that tracked HIV-1 virions carrying GFP-tagged Vpr in HeLa cells have reported a loss of ~50% of GFP-labelled puncta within the first hour of infection (46, 47). Interestingly however, unlike mature p6, Vpr has been detected in detergent-extracted HIV-1 core preparations (54, 55). As we also observed that ~10% of the Vpr from virions remains detectable in infected cells for several hours, we went on to investigate where this Vpr was located.

### Residual Vpr remains associated with CA during early infection

**Figure 6:**
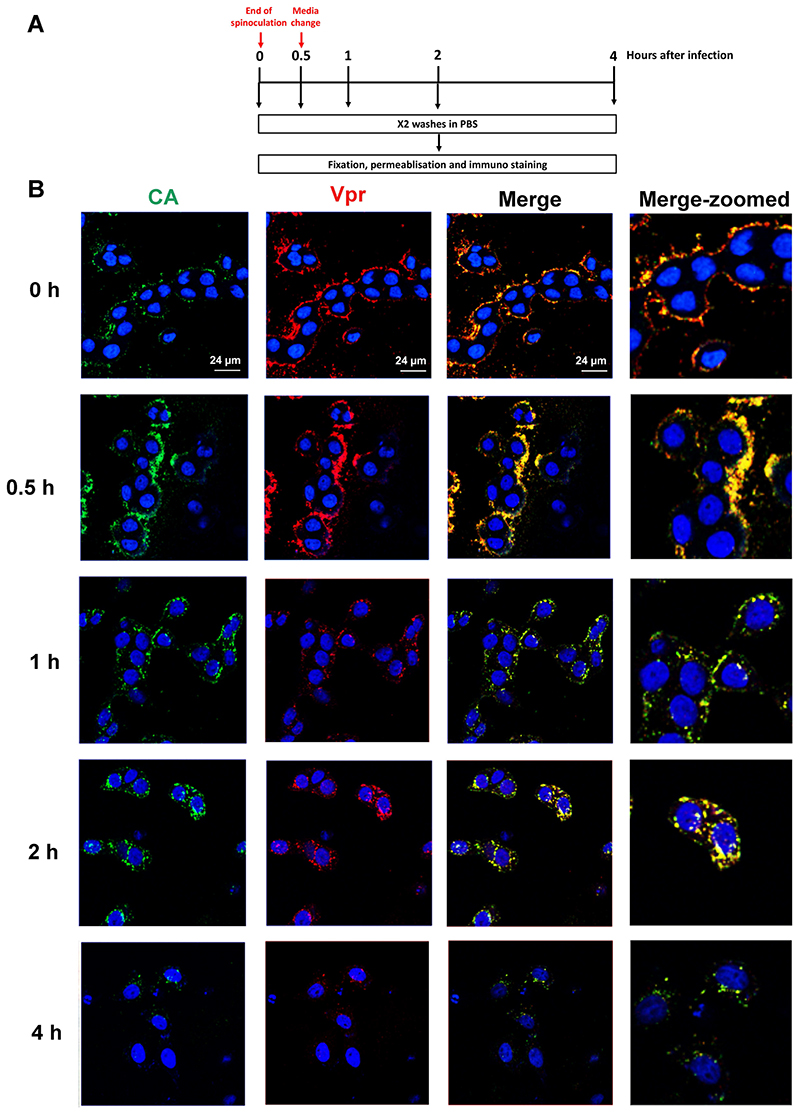
Residual Vpr remains associated with CA during early infection. Time-course assays were performed to monitor the localisation of Vpr and CA in infected cells. After infection by spinoculation with virus carrying myc-tagged p6, HeLa TZM-bl cells were either fixed immediately or incubated at 37°C for 30 min before replacement of media with fresh DMEM and subsequent fixation at different time points. Cells were washed twice with PBS prior to fixation using 4 % paraformaldehyde and methanol, then permiabilised with 0.5 % saponin and stained for CA (anti-CA, green) and Vpr (anti-Vpr, red) before confocal microscopy analysis.

To monitor the cellular localisation of the residual Vpr in host cells following infection, we next performed an immunofluorescence-based time course experiment. Following synchronised infection with pNL4-3_unc_ virus, we fixed TZM-bl cells at different time points and then co-immunostained them for Vpr and CA using anti-Vpr and anti-CA antibodies (Figure 6A). We observed clear co-localisation between Vpr and CA in the infected cells at all time points, initially at the cell periphery, presumably in virions, and after 2 h towards the nucleus, suggesting that, at least up to 4 h post-infection, some Vpr remains associated with CA (Figure 6B). Although some co-localisation could come from unfused particles in endosomes, the loss of signal intensity suggests that some protein has been lost from these complexes and that particles have fused. Furthermore, we did not observe any Vpr not associated with CA. Whilst this could be due to the limit of detection of diffuse Vpr, it does imply that free Vpr does not aggregate at a specific cellular location. Together, our data suggest that following Gag processing, ~10% Vpr molecules may dissociate from p6 and end up either encapsulated within the mature core or tethered to the outside surface of the core via alternative interactions with core proteins.

## Conclusions

Using a combination of virological and biochemical assays we have demonstrated that, unlike the MLV p12 protein, HIV-1 p6 is functionally dispensable in early replication and is rapidly lost upon host cell entry. Thus, it appears that the main roles of the p6 region of Gag are to recruit host factors required for budding and to package the accessory protein, Vpr. We have further defined the requirements for Vpr packaging, showing that two of the three previously proposed motifs in p6 are required and are functionally equivalent, and that oligomerization of Vpr contributes to packaging, likely by increasing the avidity of interactions.

Our results also suggest that ~90% of the Vpr incorporated into particles is lost very rapidly after infection, before core disassembly is thought to take place. Therefore, despite p6 being at the C-terminus of Gag, it appears that both p6 and the Vpr bound to it are primarily excluded from viral cores during maturation. Intriguingly however, the residual Vpr that remains following host cell entry seems to be retained with the virion core. How this fraction of Vpr associates with the core in the absence of p6 is unclear. It is possible that proteolytic cleavage of Gag induces dissociation of Vpr from p6 and that a small proportion of the Vpr is passively encapsidated by the CA shell. The functions of Vpr in HIV-1 replication are still in the process of been elucidated and are the subject of some debate and controversy; as well as the induction of cell cycle arrest and apoptosis, Vpr has been proposed to be involved in reverse transcription (58-61) and nuclear import (62-64). The retention of at least some Vpr with the viral CA protein provides support for functional roles for Vpr during early infection. Whether the rapid degradation or export of Vpr also has a role in viral replication remains to be elucidated, but it is surprising that so much Vpr is packaged into viral particles if most of it is dispensable. Further work is required for a more complete understanding of how Vpr contributes to HIV-1 replication.

## Materials & Methods

### Cells

HEK 293T and HeLa TZM-bl (*Bishop laboratory cell stocks*) were maintained in DMEM (*Thermo Fisher Scientific*), supplemented with 10% heat-inactivated foetal calf serum (*Biosera*) and 1% penicillin-streptomycin (*Sigma*). The cells were stored in a humidified incubator at 37°C and 5% CO_2_.

### Virus production

Replication-competent HIV-1 was produced by transfecting 293T cells with proviral plasmids derived from pNL4-3_unc_ in which *gag* and *pol* reading frames are uncoupled (a kind gift from Prof. Hans Georg Kräusslich (27)). HIV-1 p6 and Vpr mutant sequences as well as myc-tagged p6, were synthesised by GeneART (*Thermo Fisher Scientific*) and then cloned in to pNL4-3_unc_ using *XmaI/MluI* (p6) and *EcoRI/SalI* (Vpr) restriction sites. Virus-containing culture supernatants were harvested 48 h post-transfection and filtered to remove cellular debris. Viral titres were quantified by assessing reverse transcriptase (RT) activity using the Lenti RT activity kit (*Cavidi*).

### Infectivity assays

HeLa TZM-bl cells were seeded in 24-well plates (*Corning*) at densities of 5x10^4^ cells/well and infected 24 h later with equivalent RT units of WT and mutant virus. After incubation at 37°C for 48 h, cells were lysed in Tropix Lysis buffer (*Thermo Fisher Scientific*) and frozen at -20°C. To measure LacZ activity, cell lysates were mixed with Tropix galactostar reaction mixture (*Thermo Fisher Scientific*) and luminescence was measured for 1 h at 10 min intervals on a Tecan Safire plate reader.

### Immunoblotting of virus lysates

Equal volumes of WT and mutant virus were concentrated by centrifugation through a 20% (w/v) sucrose cushion for 2 h at 10,000 g, 4°C and then re-suspended in protein loading buffer and boiled. Equivalent RT units of virus lysates were next separated by SDS polyacrylamide gel electrophoresis (SDS-PAGE) and transferred onto polyvinylidene fluoride (PVDF) membranes (*Milipore*). The membranes were probed with mouse anti-CA (24-2, a kind gift from Prof. Micheal Malim), rabbit anti-Vpr (*Protein Tech*, 51143-1-AP) or goat anti-myc (*Abcam*, ab9132) primary antibodies, followed by species-specific IRDye® 800CW secondary antibodies from *LI-COR* at 1:5000 dilution. Immuno-complexes were then revealed and relevant band intensities quantified using the Li-cor Odyssey imaging and quantitation system (*LI-COR Bioscience*).

To investigate the oligomeric state of Vpr by cross-linking, equivalent RT units of WT and mutant virus were concentrated by centrifugation through sucrose and re-suspended in 1% formaldehyde in PBS for 20 min at room temperature. The cross-linking reactions were then quenched for 10 min by the addition of Tris-HCl pH 7.5 to a final concentration of 250 mM. The cross-linked virus particles were again spun through sucrose, re-suspended in protein loading buffer and then boiled, prior to analysis by immunoblotting.

### Immunoblotting of infected cell lysates

Time-course assays were performed to monitor the loss or degradation of viral p6, Vpr and CA during early host cell infection. HeLa TZM-bl cells were seeded in 6-well plates (*Corning*) at densities of 1x10^6^ cells/well and infected 24 h later with virus carrying WT or myc-tagged p6 by spinoculation (1600 g, 2 h, 16°C). The cells were incubated at 37°C for 30 min before replacement of media with fresh DMEM and returned to the incubator for harvesting at different time points. Cells from each well were washed twice in ice-cold PBS and then re-suspended in 100 μl of ice-cold RIPA lysis buffer (*Thermo Fisher Scientific*) supplemented with protease inhibitors (*Roche*). Lysis was facilitated by sonication, x7 pulses, 30 s ON and 30 s OFF, in an ice bath (Decon FS100). After sonication, 4 mM MgCl_2_ and Pierce universal nuclease (*Thermo Fisher Scientific*) were added to the lysates. After dilution with protein loading buffer, the boiled cell lysates were analysed by immunoblotting with anti-CA, anti-Vpr and anti-myc antibodies as described above.

### Immunofluorescence

Time-course assays were performed to monitor the localisation of viral Vpr and CA during early host cell infection. HeLa TZM-bl cells were seeded on 13 mm coverslips at densities of 1x10^5^ cells/well and infected 24 h later with virus by spinoculation (1600 g, 2 h, 16°C). The cells were either fixed immediately or incubated at 37°C for 30 min before replacement of media with fresh DMEM and returned to the incubator for fixing at different time points. Cells were washed twice with PBS prior to fixation using 4 % paraformaldehyde for 5 min at room temperature followed by ice-cold methanol at -20 °C for 5 min. Cells were subsequently permeablised with 0.5 % saponin (*Sigma*) in PBS for 30 min and blocked in 5% normal donkey serum (NDS, *Source Bioscience*) and 0.5% saponin in PBS for at least 1 h. Cells were then incubated with the anti-CA and anti-Vpr antibodies diluted in 1% NDS and 0.5% saponin in PBS for 1 h, at RT. Coverslips were then washed three times with PBS and incubated for another hour with anti-mouse and anti-rabbit secondary antibodies conjugated to Alexa Fluor 488 and 594 dyes (*Abcam, ab150117 and ab150064)*, diluted in 1 % NDS PBS and 0.5% saponin in PBS. Coverslips were finally washed three times and mounted in Prolong Gold media with DAPI (*Thermo Fisher Scientific*) on glass slides (*Menzel-Gläser*). Clear nail varnish was used for sealing the coverslips. Immunostained cells were visualised on a SP5 inverted confocal microscope (*Leica*) using a HCX PL APO CS 100.0x1.46 OIL (*Leica*) objective. Image analysis was performed using using FIJI (http://fiji.sc).

## Importance

The p12 protein of MLV and the p6 protein of HIV-1 are both supplementary Gag cleavage products that carry proline-rich motifs which facilitate virus budding. Importantly, p12 has also been found to be essential for early viral replication events. However, whilst Vpr, the only accessory protein packaged into HIV-1 virions, is recruited via the p6 region of Gag, the function of both mature p6 and Vpr in early replication is unclear. Here, we have systematically mutated the p6 region of gag and have studied the effects on HIV infectivity and Vpr packaging. We have also investigated what happens to p6 and Vpr during early infection. We show that, unlike p12, mature p6 is not required for early replication and that most of the mature p6, and the Vpr that it recruits, are lost rapidly upon target cell entry. This has implications for the role of Vpr in target cells.
